# Morphological Characterization and Transcriptome Analysis of New Dwarf and Narrow-Leaf (*dnl2*) Mutant in Maize

**DOI:** 10.3390/ijms23020795

**Published:** 2022-01-12

**Authors:** Lulu Han, Chenggong Jiang, Wei Zhang, Hongwu Wang, Kun Li, Xiaogang Liu, Zhifang Liu, Yujin Wu, Changling Huang, Xiaojiao Hu

**Affiliations:** 1 National Engineer Laboratory of Crop Molecular Breeding, Institute of Crop Sciences, Chinese Academy of Agricultural Sciences, Beijing 100081, China; hanlulu@genetics.ac.cn (L.H.); jiangchenggong0908@163.com (C.J.); zhangwei142327@163.com (W.Z.); wanghongwu@caas.cn (H.W.); likun01@caas.cn (K.L.); liuxiaogang2011@126.com (X.L.); liuzhifang@caas.cn (Z.L.); wuyujin@caas.cn (Y.W.); 2 National Nanfan Research Institute (Sanya), Chinese Academy of Agricultural Sciences, Sanya 572024, China

**Keywords:** maize, *dnl2* mutant, transcriptomic, phytohormones, cell wall, cell growth

## Abstract

Lodging is the primary factor limiting high yield under a high plant density. However, an optimal plant height and leaf shape can effectively decrease the lodging risk. Here we studied an ethyl methanesulfonate (EMS)-induced dwarf and a narrow-leaf mutant, *dnl2*. Gene mapping indicated that the mutant was controlled by a gene located on chromosome nine. Phenotypic and cytological observations revealed that *dnl2* showed inhibited cell growth, altered vascular bundle patterning, and disrupted secondary cell wall structure when compared with the wild-type, which could be the direct cause of the dwarf and narrow-leaf phenotype. The phytohormone levels, especially auxin and gibberellin, were significantly decreased in *dnl2* compared to the wild-type plants. Transcriptome profiling of the internodes of the *dnl2* mutant and wild-type revealed a large number of differentially expressed genes enriched in the cell wall biosynthesis, remodeling, and hormone biosynthesis and signaling pathways. Therefore, we suggest that crosstalk between hormones (the altered vascular bundle and secondary cell wall structure) may contribute to the dwarf and narrow-leaf phenotype by influencing cell growth. These results provide a foundation for *DNL2* gene cloning and further elucidation of the molecular mechanism of the regulation of plant height and leaf shape in maize.

## 1. Introduction

Maize (*Zea mays* L.) is one of the most important cereal crops in the world. Studies have demonstrated that increasing the planting density is an essential approach in order to increase per-hectare yield potential in maize [1]. However, a higher planting density can aggravate the lodging risk through increased plant height, leaf area, basal internode elongation, and center of gravity [2,3]. Plant height and leaf shape are important plant architecture traits that are closely associated with the lodging resistance, photosynthesis, and grain yield of maize [4]. The use of varieties with moderate plant height can enhance lodging resistance and improve the harvest index. With the popularization of short stature varieties during the green revolution, the yield of rice and wheat has increased sharply since the 1960s [5]. Leaf shape parameters, such as leaf length, leaf width, and leaf area, are important components of leaf morphology that affect canopy structure, photosynthetic efficiency, and wind circulation under high planting density [6]. Smaller and narrower leaves decrease shading effects on the lower leaves, enhance photosynthetically active radiation utilization, and increase maize yield potential [7]. Therefore, understanding the genetic mechanisms of maize plant height and leaf shape are important for the breeding of density-tolerant maize varieties with high grain yield.

Phytohormones, such as gibberellins (GAs), auxins (IAAs), ethylene (ETH), and brassinosteroids (BRs), play important roles in determining plant architecture traits, including plant height, leaf morphology, tiller number, and grain size [8]. For plant height, the previously characterized genes in maize are largely associated with the biosynthesis and the signal transduction of phytohormones. GAs represent a large group of cyclic diterpene compounds that are essential for stem elongation and plant height control [9]. GA synthesis, or signaling mutants, show dwarf phenotypes. The maize dwarf mutants, *anther ear1* (*an1*), *dwarf1* (*d1*), *d3*, and *d5*, have been shown to influence a different step in the biosynthesis of the GAs and are sensitive to exogenous GA application [10,11,12,13]. Two GA-insensitive dwarf mutants *D8* and *D9* were identified with altered DELLA domains, which are negative regulators of gibberellin signaling [14,15]. Auxin is an important signaling compound that is vital for plant development and growth [16]. *VT2* encodes grass-specific tryptophan aminotransferase, the mutation of which affects IAA synthesis and causes dwarfing in maize [17]. *Brachytic2* and *ZmPIN1a* regulate internode elongation by mediating the polar auxin transport in maize [18,19]. The overexpression of *ZmPIN1a* resulted in reduced plant height, ear height, and increased maize yield under high-density cultivation conditions [20]. In addition to the plant hormones GAs and IAAs, other phytohormones, such as BRs and ETH, also modulate plant height. Mutants that are deficient in BR biosynthesis or signal transduction, such as maize *na1*, *na2*, *brd1*, and the *BRASSINOSTEROID INSENSITIVE1* knockdown line, exhibit the dwarfism phenotype [21,22,23,24]. The altered C-terminus of *ZmACS7*, encoding 1-aminocyclopropane-1-carboxylic acid (ACC) synthase in ETH biosynthesis, causes a shorter stature and larger leaf angle in maize [25].

Leaf width is an important index of leaf size and is a quantitative trait that is controlled by multiple genes, including miRNA, transcription factors, and hormones [26]. Genes that are related to response factors, polar transport, and the synthesis of phytohormones are believed to be particularly important in the regulation of leaf development in rice [27]. *NAL7* (*NARROW LEAF 7*), *TDD1* (*TRYPTOPHAN DEFICIENT DWARF MUTANT 1*), and *FIB* (*FISH BONE*) are involved in auxin biosynthesis, and the reduced expression of these genes results in a narrow-leaf phenotype [28,29,30]. The auxin-deficient mutants, defective in *NAL1* (*NARROW LEAF 1)*, *NAL2/3*, *NAL21*, *OsARF11,* and *OsARF19,* which participate in auxin polar transport, distribution, and signaling, also display narrow leaves [31,32,33,34,35]. Some genes that are involved in the regulation of the gibberellin pathway, such as *PLA1, PLA2, SLR1, OsOFP2, D1,* and *GID2*, have been shown to be important in the regulation of leaf width [11,36,37,38,39]. In addition to hormones, the cellulose synthase-like (CSL) genes, which participate in hemicellulose synthesis, are important in the regulation of leaf morphology [40]. *DNL1,* which encodes cellulose synthase-like D4, functions in the M-phase to regulate cell proliferation, and the *dnl1* mutant showed a distinct narrow-leaf phenotype in rice [41]. *ZmCSLD1* is essential for plant cell division, and the *Zmcsld1* mutant exhibited narrow-organ and warty phenotypes with reduced cell sizes and cell numbers [42]. It is notable that narrow-leaf mutants commonly exhibit reduced plant height, such as *nal1-2*, *nal1-3*, *nal21*, *dnl1*, *dnl2,* and *dnl3*, implying the overlapping regulatory mechanisms of leaf size and plant height development.

In this study, we obtained the dwarf and narrow-leaf mutant *dnl2* by EMS mutagenesis. The plant height and the width of the leaves of *dnl2* differed significantly from those of the wild-type. The gene affecting the *dnl2* phenotype was located on chromosome nine. Based on the tested physiological and morphological indices, the vascular bundle patterning, secondary cell wall structure, and cell growth were altered in the leaves and internodes of *dnl2* compared to the wild-type. Moreover, some plant endogenous hormones also changed significantly. The content of GA and IAA in *dnl2* was significantly lower than that in the wild-type, while the content of ABA in *dnl2* was significantly higher than that in the wild-type. Combined with RNA-seq analysis, these results indicated that the modification of cell wall biosynthesis, phytohormone biosynthesis, and signal transduction contributes to the dwarfing and narrow-leaf phenotype of *dnl2* by influencing cell growth.

## 2. Results

### 2.1. Pleiotropic Phenotype of the Maize dnl2 Mutant

**Figure 1:**
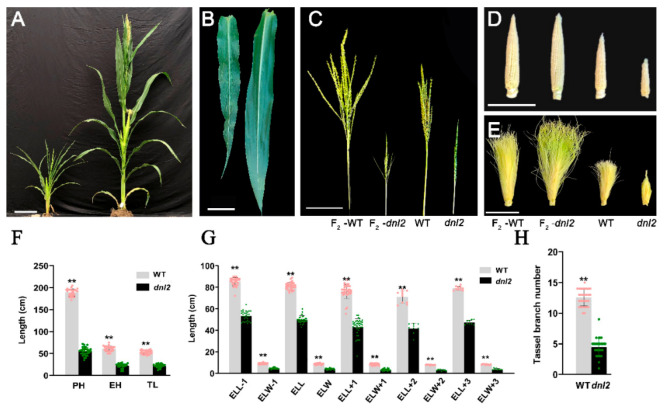
Gross morphology of WT and the *dnl2* mutant. (**A**) *dnl2* and the wild-type at tasseling stage. Bar = 20 cm. (**B**) The leaf of *dnl2* and the wild-type. Bar = 10 cm. (**C**) The tassel of *dnl2* and the wild-type. Bar = 5 cm. (**D**,**E**) The ear of *dnl2* and the wild-type. Bar = 5 cm. (**F**) Plant height, ear height, and tassel length of *dnl2* and the wild-type. PH: plant height. EH: ear height. TL: tassel length. (**G**) Measurement of the length and width of the first leaf below the uppermost ear, the leaf of the ear, and the three leaves above the ear. ELL-1, ELW-1: the length and width of the first leaf below the ear. ELL, ELW: the length and width of the leaf at the ear. ELL + 1, ELW + 1: length and width of the first leaf above the ear. (**H**) Tassel branch number. Data are means ± SD, asterisks indicate significant differences between *dnl2* and the wild-type (** *p* < 0.01).

**Figure 2:**
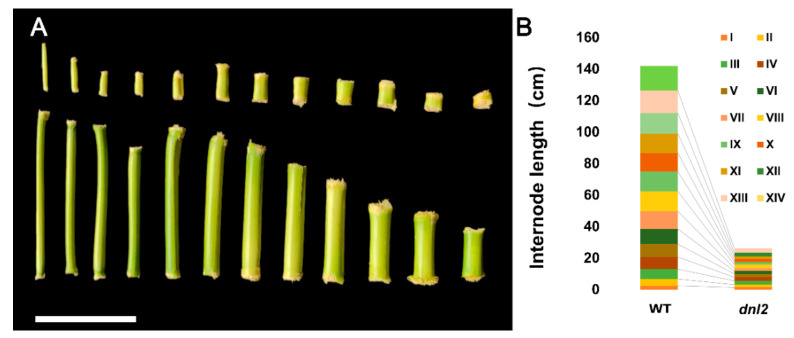
Comparison of the length of the internodes of *dnl2* and the wild-type. (**A**) Internodes of *dnl2* and the wild-type, bar = 10 cm; (**B**) Comparison of internodes length between *dnl2* and the wild-type.

The *dnl2* mutant is a recessive dwarf and narrow-leaf mutant isolated from a maize EMS-mutagenized population. When compared with its wild-type plant ‘Zheng58’, the *dnl2* mutant displayed pleiotropic developmental abnormalities, such as a short stature, narrowed and shortened leaves, and a degenerated tassel and ear (Figure 1A–E). Significant reductions in plant height and ear height were observed in the *dnl2* mutant when compared to the wild-type, with 71% and 65% reductions observed at the mature stage, respectively (Figure 1A,F). We compared the internode number and length between the wild-type and the *dnl2* mutant. Our results showed that the internode number of *dnl2* was similar to that of the wild-type, while all of the internodes were significantly shortened (Figure 2). A gradual increase in the internode length difference from the bottom to the top of the plant was observed, and the internodes above the ear showed the most significant difference, with an 80.2–85.4% reduction between *dnl2* and the wild-type. These results indicate that the dwarfing of *dnl2* was attributed to inhibited internode elongation, but not to fewer internodes. The *dnl2* mutant also had shorter and narrower leaves compared to the wild-type, and these leaf phenotypes were constantly observed in all of the leaves from the bottom to the top (Figure 1B). We measured the length and the width of the ear leaves, three leaves above the ear, and three leaves below the ear and observed a 37.3–41.6% reduction in leaf length and 49.5–62.7% reduction in leaf width in *dnl2* (Figure 1G). Reproductive development was also impacted in the mutant. The tassel, which is the terminal inflorescence, had fewer branches, and the ears, which are inflorescence branches from the main shoot, frequently failed to form (Figure 1C–E,H).

### 2.2. Inhibited Cell Growth and Altered Cell Wall Structure in dnl2 Internodes

**Figure 3:**
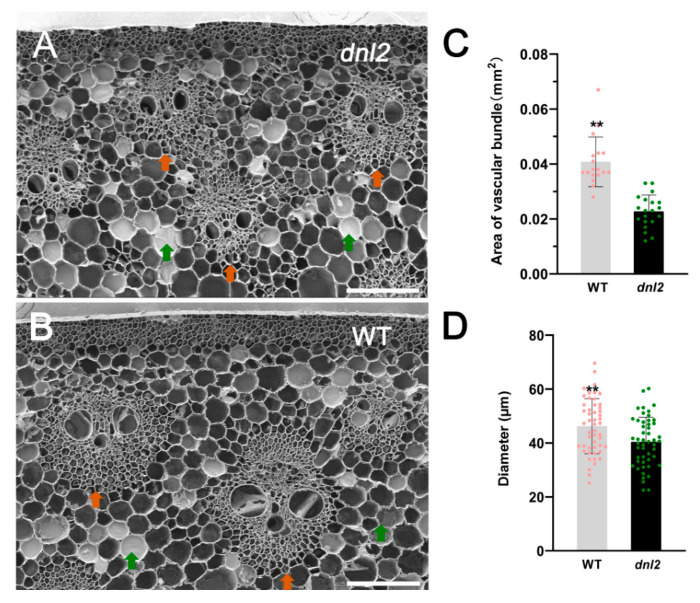
Transverse sections of the seventh internodes at the V15 stage from *dnl2* and the wild-type. (**A**,**B**) Transverse view of the vascular bundles and sclerenchyma cells of *dnl2* and the wild-type. (**C**) The area of the vascular bundles of *dnl2* and the wild-type. (**D**) The diameter of the parenchymal cells of *dnl2* and the wild-type. Orange arrowheads indicate vascular bundles and green arrowheads indicate parenchymal cells. Asterisks indicate significant differences between *dnl2* and the wild-type (** *p* < 0.01). Bars = 150 μm.

**Figure 4:**
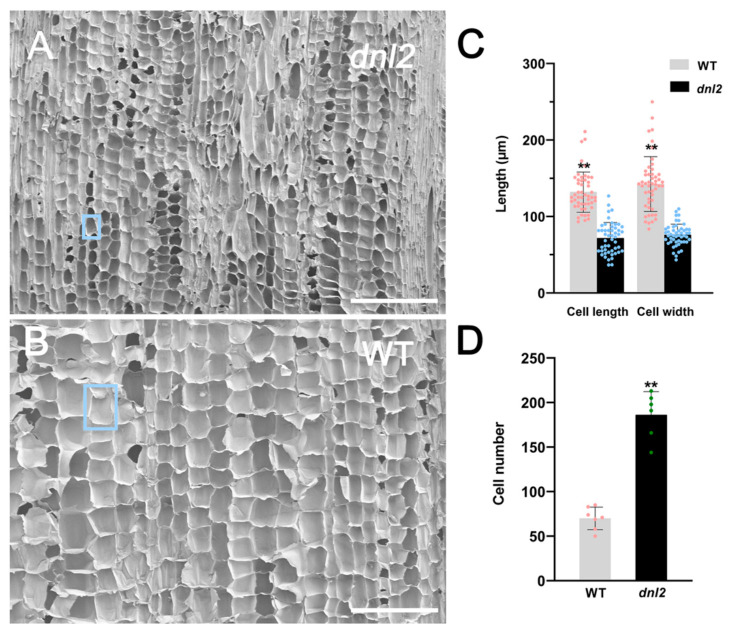
Longitudinal section of the seventh internodes at the V15 stage from *dnl2* and the wild-type. (**A**,**B**) Longitudinal view of the parenchymal cells of *dnl2* and the wild-type. (**C**) Length and width of the parenchymal cells. (**D**) Number of cells in visual field of dnl2 and the wild-type. The blue box indicates a single parenchymal cell. Asterisks indicate significant differences between dnl2 and the wild-type (** *p* < 0.01). Bars = 500 μm.

In order to determine the reason for the dwarf phenotype, we examined the anatomical features of the seventh internodes of the wild-type and the *dnl2* mutants at the 15-leaf stage through SEM. The transverse sections showed that the area of the vascular bundles near the cortex of the internode was significantly smaller in *dnl2*, reaching approximately 44.33%, compared to the wild-type (Figure 3A–C). The number of sclerenchyma cell layers around the vascular bundles were also found to be decreased in *dnl2*. The thickness of the cell walls of the sclerenchyma cells under the epidermis and surrounding xylem became thinner in *dnl2*, by 39.2% and 29.9%, respectively, compared to the wild-type, suggesting that the cell wall structure of *dnl2* was altered (Figures S1 and S2). In addition, the diameter of the parenchymal cells in *dnl2* were also significantly smaller than in the wild-type (Figure 3D). Further examination of the longitudinal sections of the internodes revealed that the parenchymal cells were irregularly arranged in *dnl2*, and the size of the cells was significantly reduced compared with the wild-type (Figure 4). The cell length and cell width of the parenchyma cells were significantly decreased by 45.5% and 46.7%, respectively, in the *dnl2* mutant (Figure 4C), while the cell number per unit area was significantly increased by 166% (Figure 4D). Taken together, these results indicate that the reduced vascular bundles size, inhibited cell growth, and decreased thickness of the sclerenchyma cell walls may be the causes of the dwarf stature of the *dnl2* mutant.

### 2.3. Narrowed Leaves and Altered Vascular Bundles Structure in dnl2

**Figure 5:**
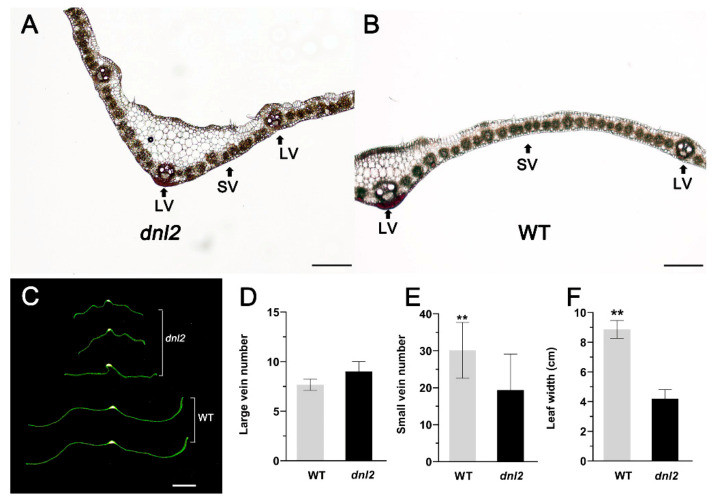
Comparison of the transverse sections of the leaves of *dnl2* and the wild-type at the mature stage. (**A**,**B**) Anatomical analysis of the transverse sections of *dnl2* and the wild-type leaves by microscope. Bar = 500 μm. LV: large vein; SV: small vein. (**C**) Transverse sections of the leaves of *dnl2* and the wild-type. Bar = 1 cm. (**D**) Large vein number of *dnl2* and the wild-type. (**E**) Small vein number of *dnl2* and the wild-type. (**F**) Leaf width of *dnl2* and the wild-type. Asterisks indicate significant differences between *dnl2* and the wild-type (** *p* < 0.01).

The structure of the vascular bundles and the number and size of the cells are the main cytological factors affecting the morphology of leaves [31,43]. In order to assess the cause of the reduction in leaf width, we observed the fourth matured leaves from the top of *dnl2* and the wild-type plants under a microscope. The transverse sections of the leaves showed that the number of small veins between two adjacent large veins was significantly decreased by 35.7% in *dnl2* compared to the wild-type, while the number of large veins in *dnl2* was comparable to the wild-type (Figure 5). The relationship between leaf narrowing and the number and size of the epidermal cells was further studied by SEM. In *dnl2*, both the number and the width of the cells along the leaf-width direction were decreased by 15.13% and 17.6%, respectively, indicating that cell division and expansion in the mutant were affected (Figure S3). Therefore, the phenotype of *dnl2* leaf narrowing was mainly affected by the decrease in the number of small vascular bundles and inhibited cell division and expansion.

### 2.4. The Cell Wall of the dnl2 Mutant Has Reduced Lignin Deposition

**Figure 6:**
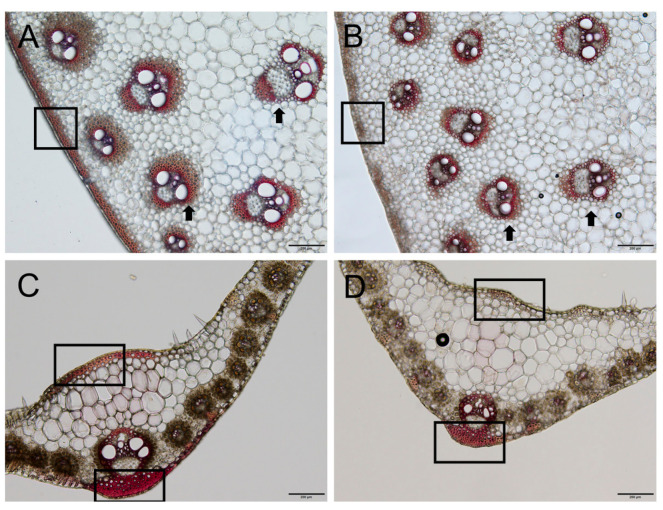
Phloroglucinol staining of lignin in the internodes and leaves. Lignin staining of the seventh internode of the wild-type (**A**) and *dnl2* (**B**) at the V15 stage. Lignin staining of the 15th leaf of the wild-type (**C**) and *dnl2* (**D**) at the V15 stage. Black arrowheads indicate vascular bundles. The black box indicates the sclerenchyma tissue. Bars = 200 μm.

Thinner secondary cell walls can be caused by insufficient cellulose, xylan, and lignin deposition. Considering that the sclerenchyma cell walls were thinner in *dnl2* than in the wild-type, we hypothesized that lignin accumulation in the sclerenchyma tissue would be greater in the wild-type than in *dnl2*. In order to test the hypothesis, we compared the deposition of lignin in the sclerenchyma tissues. The transections of *dnl2* and wild-type internodes and leaves were treated with phloroglucinol–HCl, which is a lignin-specific indicator of secondary wall thickening and observed by microscopy. The staining of the cortex, the vascular tissue near the cortex of the internodes, and the leaves showed that the layer of sclerenchyma cells of *dnl2* was significantly less than that of the wild-type (Figure 6A,B). Additionally, reduced lignification was observed in the sclerenchyma cells near the epidermis of the internodes and under the adaxial and the abaxial epidermis of the leaves (Figure 6C,D).

### 2.5. The Phytohormone Balance Was Altered in the dnl2 Mutant

**Figure 7:**
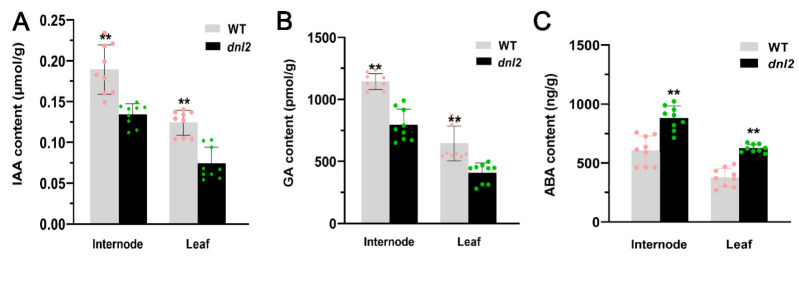
Measurement of endogenous hormones in *dnl2* and the wild-type seventh internode and 15th leaf at the V15 stage. (**A**) Measurement of IAA content. (**B**) Measurement of GA content. (**C**) Measurement of ABA content. Asterisks indicate significant differences between *dnl2* and the wild-type (** *p* < 0.01).

Phytohormones are essential for controlling plant growth and development by regulating cell proliferation and expansion. Therefore, we measured the contents of endogenous phytohormones, including GA, IAA, and ABA, of the 11th internodes and the 15th expanded leaves from the wild-type and the *dnl2* mutants at the V15 stage. The results showed that the contents of endogenous GA and IAA were significantly decreased in both the internodes and leaves of the *dnl2* mutant relative to those of the wild-type, with GA reduced by 30.62–40.03%, and IAA reduced by 29.1–40.32%, respectively (Figure 7A,B). However, the level of endogenous ABA was significantly increased by 45.56–65.57% in *dnl2* relative to the wild-type (Figure 7C). These results revealed that the phytohormone contents were disturbed in the *dnl2* mutant.

### 2.6. Genetic Analysis and Mapping of the dnl2 Mutant

**Figure 8:**
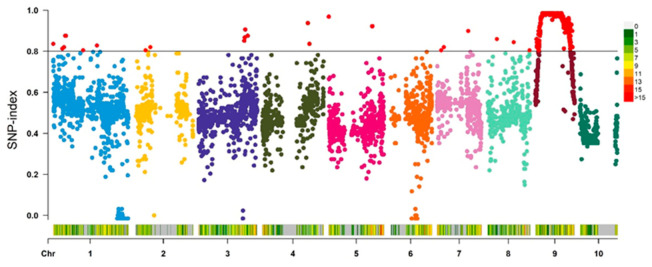
The whole-genome distribution of the SNP-index.

In order to isolate *dnl2*, the heterozygous plant (+/*dnl2*) was crossed with the ‘Mo17’ inbred line to construct an F_2_ segregation population. A total of 64 dwarf plants were sampled for preliminary mapping, and the genotypes of the samples were analyzed by genotyping by target sequencing (GBTS) technology with a 20 K marker panel. After filtering, 7357 SNP markers were identified as polymorphic between the two parents, accounting for 36.3% of the total markers. The average number of SNP markers on each chromosome was 736, and the maximum number of SNP markers on chromosome one was 1298 (Figure S4). By analyzing the variation in the SNP-index corresponding to all polymorphic SNPs at the whole-genome level, it was found that the 26.9 M–76.1 M interval on chromosome nine may be linked to the target trait (Figure 8). In order to confirm the mapping region, four new insertion/deletion (INDEL) markers were further developed to genotype another F_2_ population (dwarf plants = 400), however, no new recombination was detected at this interval. Since the region is located near the centromere and the exchange frequency is low, a larger mapping population is needed to complete the fine-mapping of the *DNL2* gene.

### 2.7. Genome-Wide Transcriptomic Analyses of dnl2 and Wild-Type Plants

**Figure 9:**
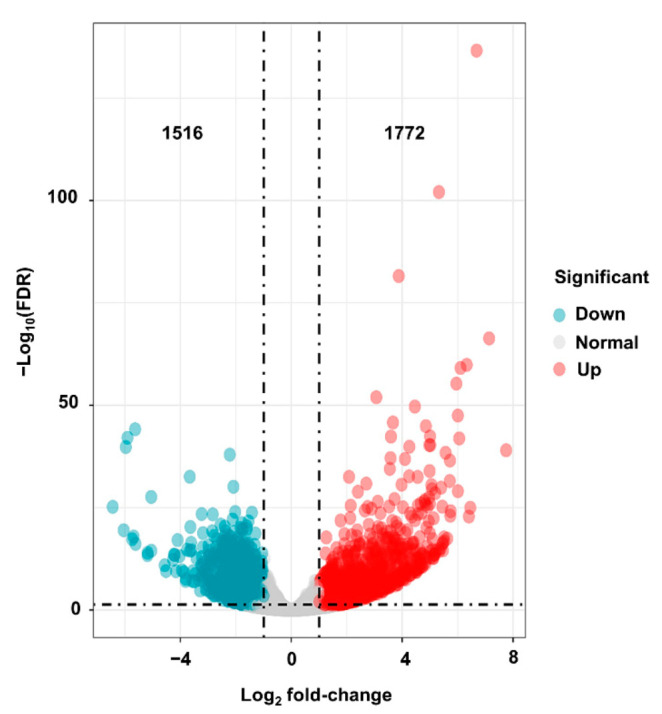
Differentially expressed genes between *dnl2* and the wild-type.

**Figure 10:**
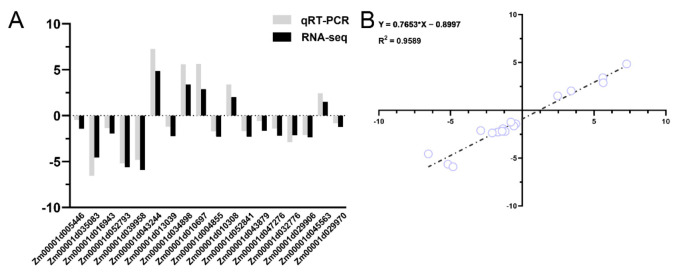
Quantitative RT-PCR validation of differentially expressed genes identified by RNA-seq. (**A**) Comparison of the relative expression between the qRT-PCR and RNA-seq results. (**B**) Correlation coefficient between the qRT-PCR results and RNA-seq results. R^2^ = 0.96.

In order to understand the transcriptome network underlying the phenotypic variations, high-throughput RNA-seq was performed for *dnl2* and wild-type plants. After quality control and filtering from raw reads, more than 21 million clean reads were generated for each sample. Approximately 78.58–86.90% of the clean reads were uniquely mapped to the maize B73 reference genome (RefGen_v4) (Table S1). A total of 27,746 and 28,652 genes with an FPKM higher than 0.1 were expressed in the wild-type and *dnl2*, respectively. A total of 3288 DEGs were identified between *dnl2* and the wild-type using the threshold FDR < 0.01 and at least a 2.0-fold expression change. Among them, 1772 genes were significantly up-regulated, and 1516 genes were significantly down-regulated, accounting for 53.89% and 46.11%, respectively, of all of the DEGs (Figure 9). The expression levels of some DEGs were evaluated by quantitative RT-PCR (qRT-PCR) in order to validate the RNA-seq data (Figure 10). Further observation of the top 10 significantly up- and down-regulated DEGs revealed that seven DEGs were annotated as cell wall-related proteins, including endo-13-beta-glucosidase, beta-galactosidase precursor, beta-D-xylosidase, polygalacturonase, and three glycosyl hydrolase family proteins, suggesting the important roles of cell wall regulation in *dnl2* development (Table S2).

**Figure 11:**
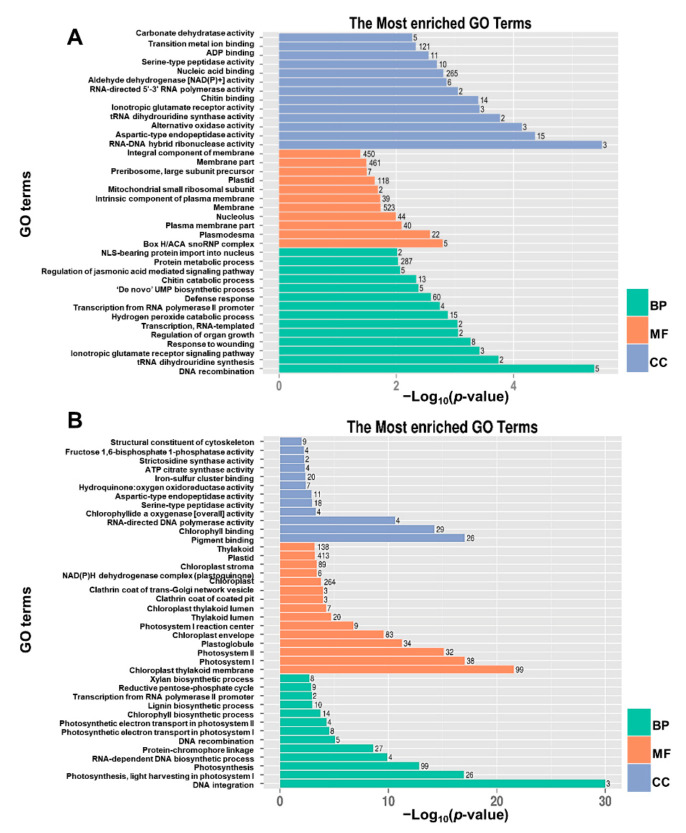
The significantly enriched GO terms of the up-regulated and down-regulated DEGs between *dnl2* and the wild-type. (**A**) The enriched BP, MF, and CC terms of the up-regulated DEGs. (**B**) The enriched BP, MF, and CC terms of the down-regulated DEGs.

**Figure 12:**
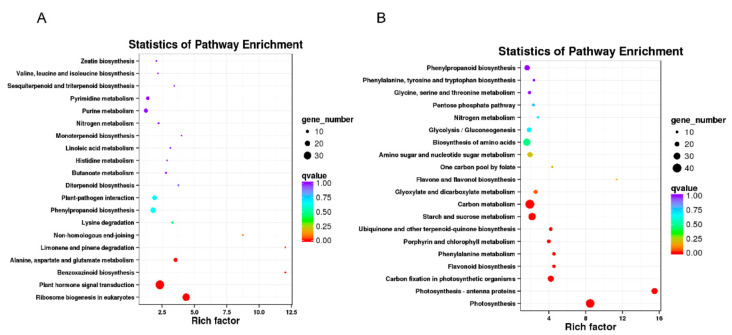
Enriched Kyoto encyclopedia of genes and genomes (KEGG) pathway enrichment of DEGs between *dnl2* and the wild-type. Enriched KEGG pathways of up-regulated DEGs (**A**) and down-regulated DEGs (**B**) in *dnl2* compared with the wild-type. The point size represents the number of genes in the pathway; the color depth indicates the *q*-value.

The GO terms and KEGG pathways were used to elucidate the functional annotations of the DEGs. The DEGs could be categorized into three main GO categories, as follows: biological process (BP), molecular function (MF), and cellular component (CC). The up-regulated DEGs were significantly overrepresented in 78 BP terms, 61 MF terms, and 19 CC terms (Figure 11A). The most significantly enriched GO terms mainly included the BP terms “protein metabolic process”, “defense response”, “hydrogen peroxide catabolic process”, and “response to wounding”; the MF terms “nucleic acid binding”, “transition metal ion binding”, and “tetrapyrrole binding”; and the CC terms “plasmodesma”, “membrane”, and “nucleolus”. For the down-regulated DEGs, 95 BP terms, 57 MF terms, and 30 CC terms were significantly enriched (Figure 11B). The “photosynthesis”, “protein-chromophore linkage”, “chlorophyll biosynthetic process”, “lignin biosynthetic process”, “xylan biosynthetic process”, and “reductive pentose-phosphate cycle” terms were the most enriched BP terms; “chlorophyll binding”, “iron-sulfur cluster binding”, and “serine-type peptidase activity” were the most enriched MF terms; and “chloroplast”, “thylakoid”, and “plastid” were the most enriched CC terms. Further KEGG enrichment analysis revealed that “ribosome biogenesis in eukaryotes”, “plant hormone signal transduction”, “benzoxazinoid biosynthesis”, “alanine, aspartate and glutamate metabolism”, and “limonene and pinene degradation” were the most overrepresented pathways in the up-regulated DEGs (Figure 12A), and “photosynthesis”, “photosynthesis-antenna proteins”, “carbon fixation in photosynthetic organisms”, “phenylalanine metabolism”, “flavonoid biosynthesis”, “porphyrin and chlorophyll metabolism”, “ubiquinone and other terpenoid-quinone biosynthesis”, “starch and sucrose metabolism”, and “carbon metabolism” were the most overrepresented pathways in the down-regulated DEGs (Figure 12B). These results indicated that the expression level of genes related to protein metabolic and hormone signaling, which were increased in the *dnl2* mutant, and the expression level of genes related to photosynthesis and cell wall biosynthesis were suppressed.

### 2.8. Altered Expression of Hormone Pathway-Related Genes in the dnl2 Mutant

**Figure 13:**
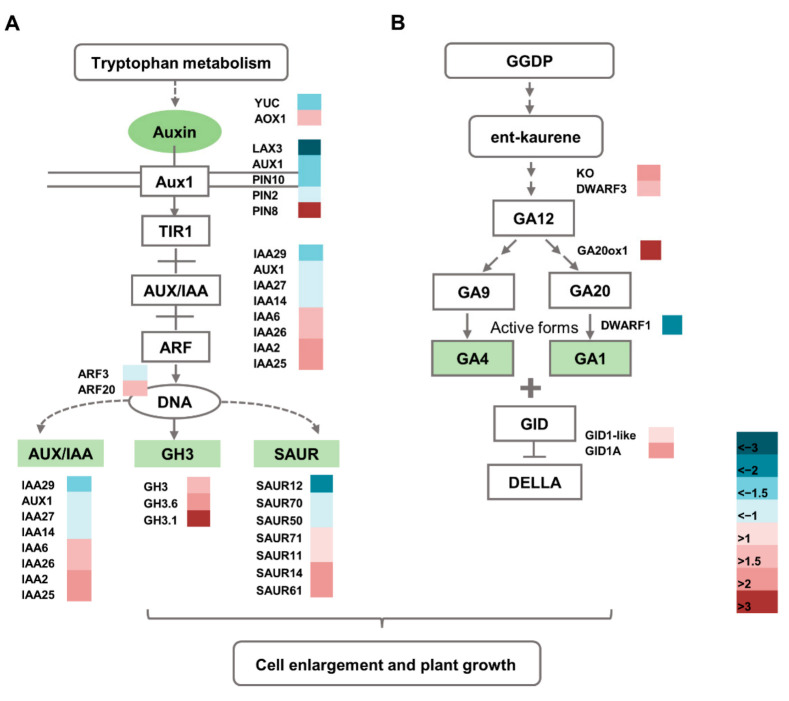
DEGs involved in phytohormone biosynthesis and signaling. (**A**) DEGs involved in auxin biosynthesis and signaling. (**B**) DEGs involved in gibberellin synthesis and signaling. On the log_2_ scale, dark blue and dark red colors represent lower and higher expression, respectively.

Phytohormones play an important role in cell elongation and plant growth. In our RNA-seq results, more than 100 DEGs related to IAA, GA, ABA, ETH, BR, and jasmonic acid (JA) synthesis and signal transduction were identified. Notably, 35 genes involved in auxin biosynthesis and signaling were differentially expressed between *dnl2* and the wild-type plants (Figure 13A and Table S3). For IAA biosynthesis, a flavin monooxygenase-like protein (*Zm00001d018652*, *YUC*), which catalyzes the last step of conversion from indole-3-pyruvate (IPyA) to IAA [44], was down-regulated by 2.75-fold in *dnl2*, while indole-3-acetaldehyde oxidase (*Zm00001d013098*, *AOX2*), which converts indole-3-acetaldehyde (IAD) into IAA [45], was up-regulated by 2.35-fold in *dnl2*. Auxin response gene families, mainly including seven *Aux/IAA* (auxin/indole acetic acid), three *GH3* (Gretchen Hagen 3), seven *SAUR* (small auxin up RNA), three *ARF* (auxin response factor), and three *PIN* (polar auxin transport), exhibited altered expression levels in *dnl2* [46,47]. *Zm00001d010697* (*GH3.6*), *Zm00001d039345* (*GH3*), and *Zm00001d043244* (*GH3.1*) belong to the *GH3* gene family, which participates in auxin homeostasis by catalyzing auxin conjugation and binding free IAA to amino acids [48], and their expression levels were increased by 2.8- to 28.6-fold in *dnl2*. *Zm00001d053004* (*AIC2*) is an auxin transporter-like protein, and its expression level in *dnl2* was decreased by 9.3-fold compared to the wild-type. The altered expression level of these genes may disturb IAA homeostasis in the *dnl2* mutant.

Gibberellins are essential regulators of internode elongation and plant height. The *DWARF1* (*Zm00001d039634*) encodes a gibberellin 3-oxidase, catalyzing the final step of bioactive GA synthesis [11]. In our RNA-seq analysis, *DWARF1* was down-regulated by 6.43-fold in *dnl2*. The *ent-kaurene oxidase* (*Zm00001d046342*, *KO*), *GA 20-oxidase* (*Zm00001d034898*, *GA20ox*), and *DWARF3* (*Zm00001d045563*) genes, which participate in the early steps of GA synthesis [12,13], were up-regulated by 2.8–to 10.5-fold in the *dnl2* mutant. GA2-oxidase (*Zm00001d017294*, *GA20ox1*), catalyzing the deactivation of bioactive GA or its precursors [49], was up-regulated in the *dnl2* mutant, which may have contributed to the decrease in endogenous GA content. As for the GA signal transduction, two *GIBBERELLIN INSENSITIVE DWARF* genes (*Zm00001d010308*, *Zm00001d038165*) encoding GA receptors were up-regulated in *dnl2* (Figure 13B and Table S4).

Furthermore, genes related to ETH, ABA, CK, BR, and JA were also found to be differentially expressed between the *dnl2* mutant and the wild-type plants (Table S5). Ethylene has been recognized as a growth inhibitor. We identified 25 ethylene-responsive transcription factors (AP2-EREBP), two reversion-to-ethylene sensitivity (RTE), and one ethylene insensitive three (EIN3) proteins exhibited increased expression levels in *dnl2*. Almost all of the genes related to ABA, CK, JA, and BR were up-regulated in *dnl2* compared to the wild-type. The altered expression level of these genes may disturb hormone synthesis and signaling homeostasis in the *dnl2* mutant, resulting in growth inhibition.

### 2.9. Altered Expression of Genes Associated with Cell Wall Development in the dnl2 Mutant

The plant cell wall not only provides mechanical strength and support, but also controls cell growth and differentiation. Since the secondary cell wall structure is altered in the *dnl2* mutant, we evaluated the expression levels of DEGs related to cell wall deposition and remodeling. Our transcriptome analysis identified more than 130 DEGs related to cell wall synthesis, remolding, and signaling, and 66.7% of the DEGs were down-regulated in the *dnl2* mutant compared to the wild-type plants. These DEGs were further divided into several classes according to the functional annotation, and most of them were classified as secondary cell wall-related.

**Figure 14:**
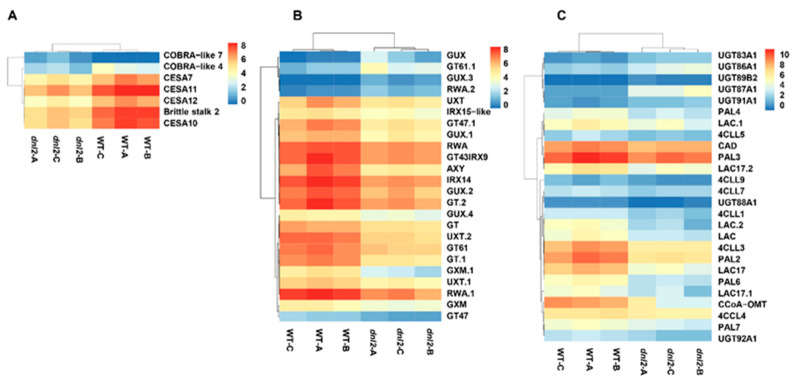
Heatmap of cell wall related DEGs. (**A**) Heatmap of DEGs involved in cellulose synthesis of the secondary cell wall. (**B**) Heatmap of DEGs participated in xylan synthesis. (**C**) Heatmap of DEGs participated in lignin synthesis.

Cellulose synthase (CesA) participates in cellulose synthesis during primary and secondary cell wall deposition. The maize genome contains at least 12 *CesA* genes, and *CesA1*–*9* are believed to be involved in primary cell wall formation, while *CesA10*–*12* are involved in secondary wall deposition [50]. Our RNA-seq results revealed that four *CesA* genes, including *CesA7* (*Zm00001d005775*), *CesA10* (*Zm00001d032776*), *CesA11* (*Zm00001d043477*), and *CesA12* (*Zm00001d020531*), were all down-regulated in the *dnl2* mutant. The *Brittle stalk 2* (*Zm00001d047276*), and another COBRA-like protein (*Zm00001d022082*), which participate in the cellulose synthesis of the secondary cell wall [51], also exhibited decreased expression levels in *dnl2* (Figure 14A and Table S6). Xylan is the second-most abundant polysaccharide in plant secondary walls [52]. Several enzymes have been implicated in xylan synthesis and substitution, such as glycosyl transferase (GTs) gene families, glucuronosyltransferase (GUXs), glucuronoxylan 4-O-methyltransferase (GXMs), and reduced wall acetylation (RWAS) [53]. Ten GTs, five GUXs, two GXMs, three UDP-xylose transporters (UXT), and three RWAs were identified to have altered expression levels in *dnl2*, and 80% of them were down-regulated, suggesting that xylan synthesis may be affected in the mutant. *Zm00001d011959* and *Zm00001d028980* are GT47 family genes, which are required for synthesizing the xylan backbone, and their expression level in *dnl2* decreased by 2.5–3.7-fold compared to the wild-type. *Zm00001d010976* and *Zm00001d036543*, which belong to the GT43 family and encode IRX9 and IRX14 respectively, were also down-regulated in *dnl2* (Figure 14B and Table S7). Lignin is an abundant biopolymer of phenylpropanoid monomers and is critical for plant structure and strength [53]. In our study, the expression of five phenylalanine ammonia-lyase (PAL), six 4-coumarate-CoA ligase (4CL), six laccase (LAC), and one caffeoyl-CoA 3-O-methyltransferase (CCoA-OMT) genes involved in lignin biosynthesis were all decreased in *dnl2* (Figure 14C and Table S8). In addition, the genes involved in pectin, hydroxyproline-rich glycoproteins (HRGPs), and APG protein synthesis, such as β-1, 4-galactosyltransferases, extensions (EXTs), and fasciclin-like arabinogalactan, were all down-regulated (Table S9). The decreased expression of these cell wall synthesis-related genes may greatly affect the cell wall structure in the *dnl2* mutant, leading to stunted growth.

Growth begins with cell wall loosening [54]. During the elongation phase of cell growth, a number of enzymes and proteins, such as expansins (EXP), beta-glucosidase (BGL), xyloglucan endotransglycosylases/hydrolase (XETs/XTHs), and endo-(1,4)-β-d-glucanase (EG), are believed to mediate the wall loosening process [54], and 48 DEGs involved in this process were identified in our study (Table S10). Eight *EXPANSIONS*, which are primary agents in regulating cell wall enlargement, had changed expression levels in *dnl2*, five of which were down-regulated and three of which were up-regulated. Beta-glucosidase is a component of cellulose enzymes that is essential for the complete hydrolysis of cellulose into glucose [55]. Seventeen DEGs encoding beta-glucosidase were discovered in *dnl2*, and two of them were up-regulated by more than 128-fold compared to the wild-type. Furthermore, genes encoding endoglucanase, xyloglucan endotransglycosylases/hydrolase, β-xylanase, β-galactosidase, and β-D-xylosidase were also identified with altered expression levels in the mutant.

## 3. Discussion

The *dnl2* mutant is a recessive mutant caused by EMS mutagenesis that displays various developmental defects, with a short stature and narrowed leaves being the two most obvious features. In this study, we combined phenotypic and cytological observations, physiological and biochemical analyses, and transcriptome sequencing in order to explore the possible regulation mechanism underlying the mutant phenotype of *dnl2*. Our results demonstrated that the vascular bundle patterning, cell wall structure, and cell growth were altered in *dnl2* internodes and leaves compared with the wild-type plants, which could be the direct cause of the dwarf and narrow-leaf phenotype (Figure 3, Figure 4 and Figure 5). The phytohormone levels were also altered in *dnl2*, and the IAA and GA contents were particularly significantly decreased compared to the wild-type plants (Figure 7). Defects in phytohormone synthesis and response can significantly disturb cell division, cell expansion, and vascular development in *dnl2*. Genome-wide transcriptome profiling of the internodes of the *dnl2* mutant and wild-type revealed a large number of DEGs enriched in the cell wall biosynthesis, remodeling, and hormone biosynthesis and signaling pathways. These results further elucidated the transcriptional regulation underling the mutant phenotype of *dnl2*.

### 3.1. Inhibited Cell Division and Expansion Result in the Dwarf and Narrow-Leaf Phenotypic of dnl2

Plant organ shape and size are precisely controlled by localized cell division and subsequent cell expansion during plant growth [56]. Extensive studies indicate that impaired mitosis, cell elongation, and expansion could result in a reduction in plant height, leaf area, and grain yield [57,58,59]. In rice, *Dwarf1* (*D1*) encodes the α-subunit of the GTP-binding protein, which regulates cell division, promotes internode elongation, and influences plant height development [11]. The *stemless dwarf 1* (*STD1*) encodes a phragmoplast-associated kinesin-related protein and has a fundamental role in cell division. The *std1* mutant exhibited no differentiation of the node and internode organs, abnormal cell shapes, and a reduced cell division rate [60]. The *Narrow leaf1* (NAL1) gene functions in cell division rather than cell elongation, and the *nal1* mutant exhibited a dwarf and narrow-leaf phenotype with defective cell division [31]. In maize, *Narrow Odd Dwarf* (*NOD*) plays a cell-autonomous function. The *nod* mutants have smaller organs due to fewer and smaller cells [61]. In our study, the maize *dnl2* mutant exhibited inhibited internode elongation and reduced leaf size. Internode elongation is driven by cell division in the intercalary meristem, followed by cell expansion in the elongation zone. A comparison of longitudinal sections taken from the *dnl2* and wild-type internodes revealed that the parenchymal cells were irregularly shaped in *dnl2*, and both the cell length and width were significantly reduced compared to the wild-type (Figure 4), which suggested that cell elongation growth in the *dnl2* internodes was suppressed. However, the cell number per unit was found to be significantly increased in *dnl2*, which could be an induced compensation phenomenon for the reduction in cell size. In the leaves, both the cell number and the cell width along the width direction of the leaf blade were decreased in *dnl2* compared to the wild-type, while no significant change was observed in cell length (Figure 5). These results implied that the *DNL2* gene has essential roles in cell proliferation and expansion. The reduced cell size and cell number are the major causes of the dwarf and narrow-leaf phenotype of *dnl2*.

Vascular bundle development is also an important determinant of plant height and leaf morphology. In rice, several mutants with reduced plant height and leaf width similar to that of *dnl2* have been reported. Cross-section examination of the leaf blades of these mutants, such as *nal1*, *nal7*, *nrl1,* and *tdd1*, have demonstrated that narrow leaves mainly resulted from a defect in cell proliferation and a reduced number of vascular bundles [28,29,31,62]. In *dnl2*, altered vascular bundle patterning in the internodes and leaves was also observed. Compared with wild-type plants, the area of the vascular bundles was much smaller in the shortened internodes of *dnl2* (Figure 3), and the number of small veins was significantly reduced in the leaves of *dnl2* (Figure 5). The changed vascular bundle patterning in the internodes and leaves of *dnl2* may be caused by either earlier defects in the recruitment of founder cells, or later defects in the differentiation of cells into vascular tissues, which suggested that the *DNL2* gene was also crucial for determining vascular cell identity.

### 3.2. Altered Cell Wall Structure and Transcriptional Regulation Result in Defective Cell Growth in dnl2

Cell wall biosynthesis is important for regulating cell shape and size in the process of plant cell growth [63]. The change of vacuole turgor pressure is the main driving force in plant cell growth, and cell growth also depends on the synthesis and remodeling of cell wall polysaccharides [64]. In rice, the *narrow leaf and dwarf1* (*nd1*) mutant exhibits significant growth inhibition due to suppressed cell division. Map-based cloning has revealed that the *ND1* gene encodes OsCSLD4, which plays an important role in modifying the cell wall structure. The expression analysis revealed that OsCSLD4 is specifically expressed in M-phase cells in order to regulate cell proliferation [65]. *ZmCSLD1* encodes an enzyme in cell wall biosynthesis and controls organ size by altering cell division. The inactivation of *ZmCSLD1* also results in the narrow leaf and stunted phenotype mainly due to the decrease in cell number [42]. In our study, the thickness of the secondary cell wall of the vascular bundles in both the internodes and the leaves of *dnl2* was significantly reduced compared to the wild-type (Figure 4 and Figure 5). The histochemical staining results also indicated reduced lignin deposition in the secondary cell wall of *dnl2* (Figure 6). The altered cell wall structure may be related to the inhibited cell division and elongation.

During rapid cell growth, the development of new cell wall polymers relies on a large amount of cellulose and hemicellulose deposition, which is manipulated by the active expression of cell wall-related genes [66,67]. Transcriptome comparison between *dnl2* and the wild-type showed that 66.7% of the 130 DEGs that are related to cell wall deposition and remodeling were down-regulated in *dnl2* compared with the wild-type, especially the DEGs involved in secondary wall deposition (Figure 14). For example, *CesA10*, *CesA11*, *CesA12,* and *Brittle stalk 2*, which are abundant in the vascular bundles and are associated with secondary wall cellulose synthesis, were down-regulated by 2.2–7.2-fold (Figure 14A). Twenty DEGs belong to GTs, GUXs, GXMs, and RWAS families, which participate in xylan synthesis and substitution, were also down-regulated (Figure 14 B). Additionally, 21 DEGs related to lignin synthesis were down-regulated, such as two PALs (*Zm00001d003016*, *Zm00001d003015*), which are the key enzymes of the phenylpropanoid pathway and exhibited 6.2–7.1-fold decreased expression levels. CCoAOMT (*Zm00001d052841*), which is involved in an alternative methylation pathway of lignin biosynthesis, was also decreased in expression by 4.8-fold (Figure 14C). These expression changes explain the thinner secondary cell wall and decreased deposition of lignin around the vascular bundles and under the epidermis of *dnl2* internodes and leaves.

### 3.3. Plant Hormones May Participate in the Regulation of Cell Growth and Vascular Patterning in dnl2

Plant growth and development are tightly regulated by phytohormones, such as auxin and gibberellin [68]. Auxin plays a pivotal role in regulating cell wall remodeling and overall cell growth [69]. Numerous mutants impaired in auxin synthesis or signaling exhibit overall dwarfism, defects in tropisms, and alterations in organ morphology [70]. In maize, loss-of-function of *VANISHING TASSEL* (*VT2*), which is a grass-specific IAA biosynthetic enzyme in the IPA pathway, shows shorter inflorescences and plant height due to defects in cell elongation [17]. The reduction in IAA levels gives rise to pleiotropic organ malformation together with a severe narrow-leaf phenotype in rice. The *narrow leaf7* (*nal7*) mutant, which has a mutation in YUCCA8 (YUC8) that is involved in auxin synthesis, produces narrow and curly leaves throughout development [28]. *NAL1* regulates the polar transport of auxin and modulates leaf size by affecting vein patterning and cell division [31]. Recent studies have shown that *NAL2/3* not only regulates auxin distribution, but also has a negative feedback effect on gibberellin biosynthesis. It is suggested that *NAL2/3* may regulate leaf size through the crosstalk between GA and auxin [32]. In both the *nal1* and *nal2/3* mutants, the number of small veins in the leaves is significantly reduced, whereas the number of large veins is only slightly reduced compared to the wild-type. In our study, *dnl2* showed a significant decrease in the number of small veins compared with the wild-type plants. The GA and IAA contents were significantly decreased in both the internodes and the leaves of *dnl2* relative to those of the wild-type (Figure 7). Therefore, we speculate that *dnl2* has similar regulatory mechanisms as *nal1* and *nal2/3*, caused by the crosstalk of IAA and GA. Our transcriptome results revealed that many genes involved in IAA and GA biosynthesis and signaling were differentially expressed between *dnl2* and the wild-type plant (Figure 13). Flavin monooxygenase-like protein, which catalyzes the last step of conversion of IPyA to IAA, was down-regulated by 2.75-fold in *dnl2*. *DWARF1*, which encodes a gibberellin 3-oxidase that catalyzes the final step of bioactive GA synthesis, was also down-regulated by 6.43-fold in *dnl2*. Down-regulation of the expression of these genes could be the cause of the decreased IAA and GA contents in *dnl2*. Furthermore, auxin response gene families, such as Aux/IAA, GH3, SAUR, ARF, and PIN, and GA receptors exhibited altered expression in *dnl2*. Therefore, we hypothesized that the dwarfing mechanism of *dnl2* is caused by the crosstalk between hormones, such as GA and IAA, which regulates the synthesis of the plant secondary cell wall, thus affecting the elongation of plant cells.

## 4. Materials and Methods

### 4.1. Plant Materials and Phenotypic Analysis

The pollen of the maize inbred line ‘Zheng58’ was collected and mutagenized with ethyl methanesulfonate (EMS), and the resulting pollen was applied to ‘Zheng58’ female ears to generate M_1_ progeny. A large number of M_1_ seeds were planted and self-pollinated to produce the M_2_ population, among which a dwarf and narrow-leaf mutant was identified and named *dnl2*. The *dnl2* with stable inheritance was obtained by continuous selfing and screening. For phenotypic analysis, the *dnl2* mutant and normal siblings (wild-type) of the same M_5_ family were used. All materials were single seeded and nursed with standard agricultural cultivation. The method of measuring plant height and ear height was from the ground to the tassel top, and from the ground to the top ear-bearing node, respectively. Leaf angle was the inclination between the internode and leaf blade midrib. The length of the leaves above the top ear was measured along the midrib, from the ligule to the tip, and the width was measured at the midpoint of the length. Analysis was performed on 10 individual plants for each genotype.

### 4.2. Gene Preliminary Mapping

Due to the poor fertility of the *dnl2* mutant, the F_1_ population was obtained by crossing hybrid plants (+/*dnl2*) of the M_4_ progeny with the maize inbred line ‘Mo17‘. The F_2_ population obtained from F_1_ selfing was used for genetic analysis and gene mapping. Dwarf mutants were randomly selected from the F_2_ population, and the genomic DNA of 67 mutant plants and their parents was extracted using the CTAB method [71]. The genotypes were assessed via genotyping by target sequencing (GBTS) with a 20 K single nucleotide polymorphism (SNP) panel [72]. After removing the non-polymorphic and low-quality markers, the genotype frequencies (SNP-index) of each polymorphic SNP marker were calculated. The SNP index represents the frequencies of mutant alleles in the population. The closer the SNP-index is to 1, the closer linkage between the marker and the target gene [73].

### 4.3. Measurement of Endogenous Phytohormones

Endogenous GA, ABA, and IAA were measured using a plant enzyme-linked immunosorbent assay (ELISA) kit (Shanghai Enzyme-linked Biotechnology Co., Ltd., Shanghai, China) according to the producer’s instructions. In order to measure the concentration of GA3, ABA, and IAA, the 15th expanded leaves and the 11th internode of *dnl2* and WT at the V15 stage were ground in liquid nitrogen. The GA3, ABA, and IAA ELISA kit includes a set of standard samples. The standard samples were assayed at the same time as the plant samples which allowed the operator to produce a standard curve of optical density (O.D.) versus GA3, ABA, and IAA concentration. The concentrations of GA3, ABA, and IAA in the samples were then determined by comparing the O.D. of the samples to the standard curve [74].

### 4.4. Histochemical Staining

The seventh internode from the bottom of the stem and the 15th leaf at the tasseling stage were sampled from *dnl2* and the wild-type. Three biological replicates were assessed. The samples were embedded in 3% agar for the observation of cells and tissues via light microscopy. Transverse sections (100 μm) were produced using a vibratome (Leica VT 1000 S). Following staining with phloroglucinol HCl, the images were collected with an Olympus BX53 microscope under white light.

### 4.5. Scanning Electron Microscopy (SEM)

The seventh internodes and the 15th leaf of the wild-type and *dnl2* plants at the V15 stage (15 expanded leaves) were used for SEM observations. The internodes and leaves were cut into 2-mm longitudinal and transverse sections and fixed in FAA (formalin: acetic acid: 70% ethanol, 1:1:18, *v*/*v*/*v*). The fixed material was dehydrated in an ethanol gradient series (70%, 80%, 95%, and 100% ethanol) and then treated with isoamyl acetate for 15 min twice to replace the remaining ethanol and subjected to critical point drying (Hitachi Regulus8100). The samples were then coated with Pt particles and analyzed under a scanning electron microscope SU8020 (Hitachi, Tokyo, Japan). ImageJ software was used to measure the size of the cell, the number of cells, and the area of the vascular bundles.

### 4.6. RNA-seq Library Construction and Sequencing

For RNA-seq analysis, the seventh internodes of the wild-type and mutant plants at the V15 stage were harvested and frozen in liquid nitrogen. Three biological replicates for each genotype and three pooled samples for each replicate were tested in this study. Total RNA was extracted with the Transzol UP kit (Beijing Transgen Biotechnology Co., Ltd., Beijing, China), and the RNA concentration, purity, and integrity were examined using advanced molecular biology equipment. A total amount of 1 μg qualified RNA per sample was used as input material for the RNA sample preparations. Based on the manufacturer’s instructions, sequencing libraries were generated using the NEBNext UltraTM RNA library prep kit for Illumina (New England Biolabs, Ipswich, MA, USA), and index codes were added to attribute sequences to each sample. The library quality was assessed on an Agilent Bioanalyzer 2100 system. The clustering of the index-coded samples was performed on a cBot cluster generation system using a TruSeq PE cluster kit v4-cBot-HS (Illumina, San Diego, CA, USA) according to the manufacturer’s instructions. After cluster generation, the library preparations were sequenced on an Illumina HiSeq2500, and 125 bp paired-end reads were generated.

### 4.7. Sequence Mapping, Expression Quantification, and Differential Expression Analysis

After removing reads containing adapters or poly-N and low-quality reads (*q*-value ≤ 10) from the raw data, the paired-end clean reads were aligned to the B73 reference genome (RefGen_v4) using the default parameters of HISAT2 software. The reference genome and gene model annotation files were downloaded from the genome website (http://ensembl.gramene.org/Zea_mays/Info/Index) [Accessed: 6 December 2020] directly. The read count numbers of fragments per kilobases per million reads (FPKM) were converted using Stringtie v2.1.0 software. The differential expression analysis between the wild-type and the *dnl2* mutant was performed was performed using DESeq2. The resulting *p*-values were adjusted using the Benjamini and Hochberg’s approach for controlling the false discovery rate (FDR). Genes with Log_2_ fold-change (Log_2_FC) ≥ 1 (up-regulated) or Log_2_FC ≤ −1 (down-regulated) and FDR < 0.01 were considered as differentially expressed genes (DEGs).

### 4.8. Gene Ontology (GO) and Pathway Enrichment Analysis

GO enrichment analysis of the DEGs was implemented using the GOseq R package and GO terms with corrected *p*-values < 0.05 were considered to be significantly enriched by DEGs. KOBAS software was used to test the statistical enrichment of DEGs in Kyoto Encyclopedia Genes and Genomes (KEGG) pathways.

### 4.9. Quantitative Real-Time PCR (qRT-PCR) Validation of the DEGs

The expression levels of some DEGs were evaluated by qRT-PCR to validate the RNA-seq data. The specific primers for qRT-PCR are provided in Table S11, *tubulin* was used as an internal control in the qRT-PCR. The reaction was performed in a 96-well plate on a CFX96 real-time PCR detection system (Bio-Rad, Hercules, CA, USA) using TB Green II Premix Ex Taq (RR820A; TaKaRa Biotechnology Co., Ltd., Dalian, China) using the thermal cycling parameters (30 s at 95 °C, 40 cycles of 5 s at 95 °C and 30 s at 60 °C; dissociation curve: 65 °C to 95 °C, increment 0.5 °C, for 5 s). The relative expression level of the selected DEGs was calculated with the 2^−ΔΔCT^ method [75]. The reaction was carried out using three biological replicates with three technical replicates.

## 5. Conclusions

In this study, we characterized a recessive maize mutant, *dnl2*, which exhibited short internodes, narrow leaves, and various developmental defects. Genetic analysis suggested the *DNL2* was located near the centromere of chromosome nine. The phenotypic, cytological, and biochemical comparison between the *dnl2* and the wild-type mutants revealed that the cell growth, vascular bundle patterning, cell wall structure, and phytohormone contents were altered in the internodes and leaves of *dnl2*, which were the main causes of the defective phenotype. The transcriptome analysis further proved that the crucial genes involved in cell wall development, phytohormone synthesis, and signaling were differentially expressed between the *dnl2* and wild-type plants. Our study provides important clues for the further elucidation of the molecular mechanism of the regulation of plant height and leaf shape in maize.

## Data Availability

The data presented in this study are available in this article and Supplementary Materials.

## Supplementary Materials

The following supporting information can be downloaded at: https://www.mdpi.com/article/10.3390/ijms23020795/s1.
