# Supplementary material for: Morphological Characterization and Transcriptome Analysis of New Dwarf and Narrow-Leaf (dnl2) Mutant in Maize
Source: Int J Mol Sci. 2022 Jan 12;23(2):795. doi: 10.3390/ijms23020795 (PMC8775757; doi:10.3390/ijms23020795)
Supplement: Supplementary file 1 [file ijms-23-00795-s001.zip › ijms-1538003-supplementary.pdf]

International Journal of
Molecular Sciences

MDPI

Supplementary figures and tables

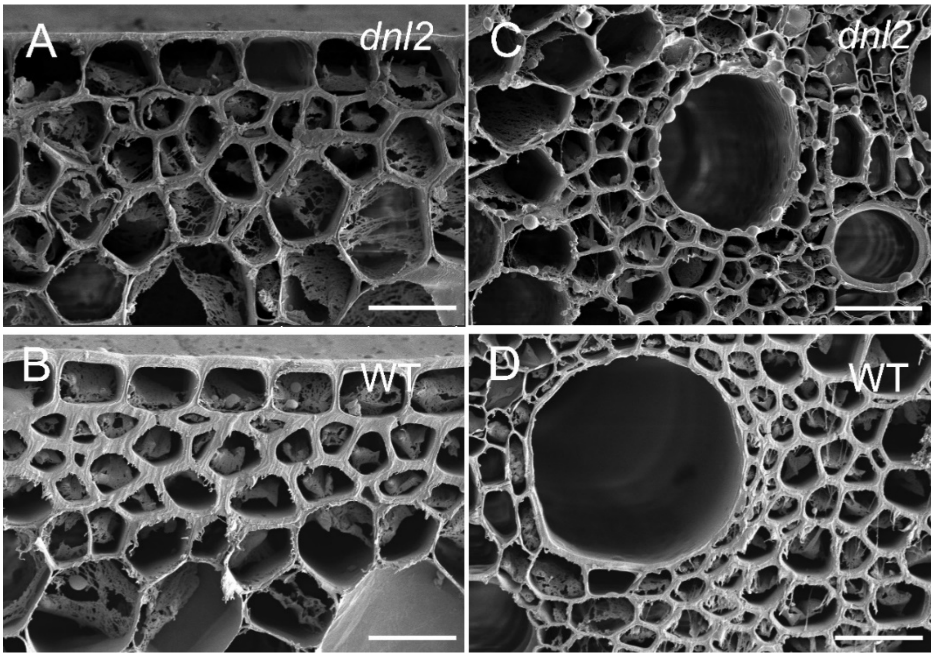

Figure S1. Scanning electron microscopy examination of the transverse sections of seventh internodes at the V15 stage from *dnl2* and the wild-type**.** (**A**,**B**) Transverse view of the thickness of cell wall of the sclerenchyma cells under epidermis of *dnl2* and WT. Bars = 20 µm (**C**,**D**) Transverse view of the thickness of cell wall of the sclerenchyma cells surround xylem of *dnl2* and WT. Bars = 25 µm.

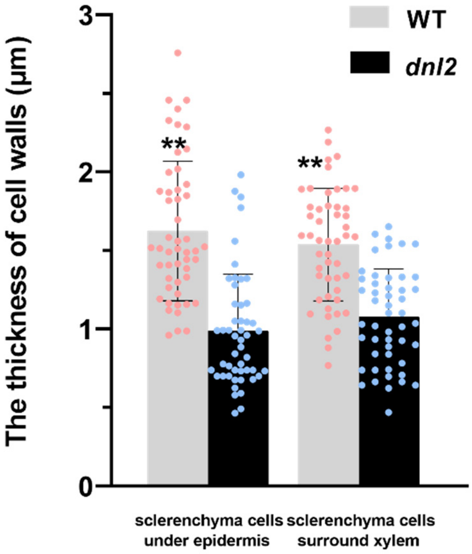

Figure S2. The thickness of cell walls of the sclerenchyma cells under epidermis and surround xylem of the seventh internodes at the V15 stage from *dnl2* and the wild-type. Asterisks indicate significant differences between *dnl2* and the wild-type (** $P < 0.01$).

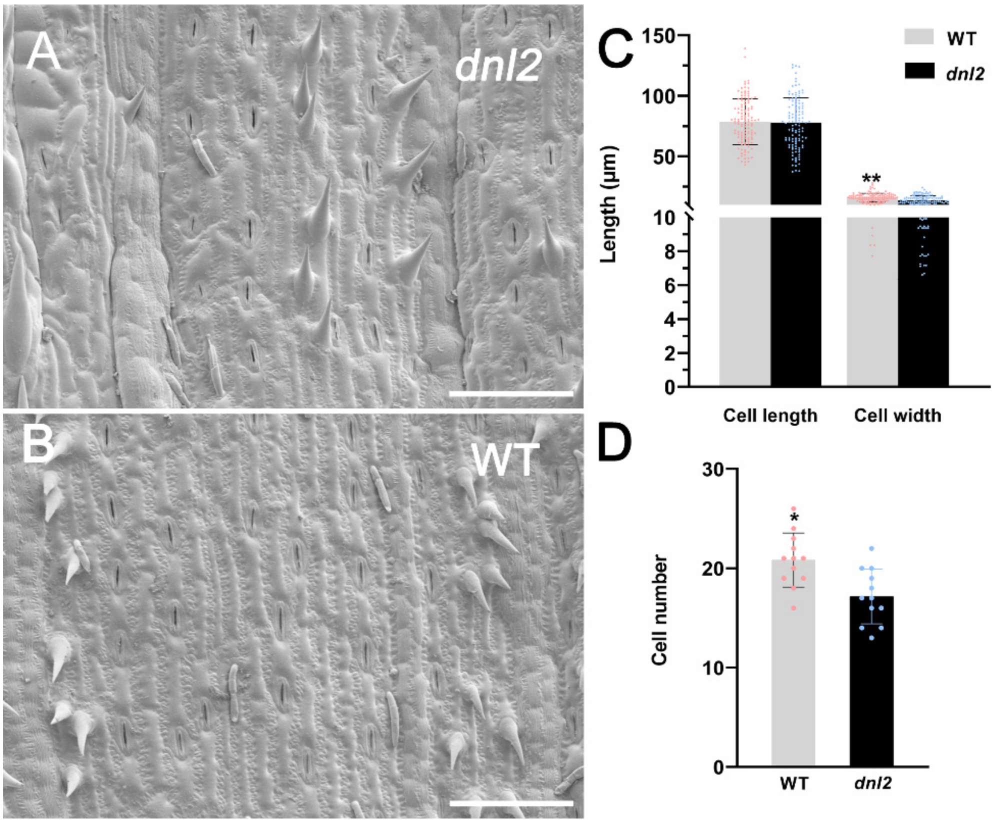

Figure S3. Scanning electron microscopy examination of the leaf epidermis at the V15 stage from *dnl2* and the wild-type. (**A**,**B**) SEM examination of the epidermis of *dnl2* and the wild-type. (**C**) Length and width of the Epidermis cells, (**D**) The number of cells in the visual field of *dnl2* and the wild-type. Asterisks indicate significant differences between *dnl2* and the wild-type (* $P < 0.05$; ** $P < 0.01$). Bars = 150 μm.

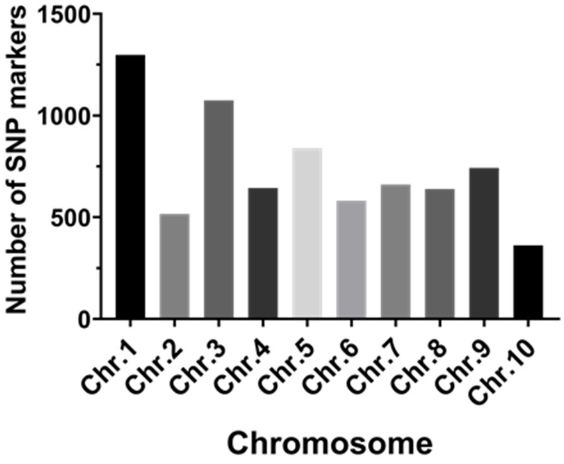

Figure S4. The average number of SNP markers on each chromosome.

Table S1. Mapping statistics for quality filtered reads of WT and *dnl2*.

Samples	Total Reads	Mapped Reads	Unique Mapped Reads	Multiple Map Reads
dnl2-A	46,300,798	40,117,084 (86.64%)	39,039,065 (84.32%)	1,078,019 (2.33%)
dnl2-B	47,645,610	39,789,068 (83.51%)	38,672,790 (81.17%)	1,116,278 (2.34%)
dnl2-C	42,456,506	34,422,597 (81.08%)	33,362,139 (78.58%)	1,060,458 (2.50%)

WT-A	56,330,838	50,196,806 (89.11%)	48,931,934 (86.87%)	1,264,872 (2.25%)
WT-B	43,624,362	38,320,895 (87.84%)	37,322,612 (85.55%)	998,283 (2.29%)
WT-C	46,313,408	41,286,656 (89.15%)	40,245,471 (86.90%)	1,041,185 (2.25%)

A, B, C indicated three biological replicates.

Table S2. The top10 significantly up- and down-regulated DEGs.

**Gene ID**	**Protein**	**FDR**	**Log_2FC**	**Regulated**
Zm00001d035178	Indole-2-monooxygenase-like CYP71A26	3.21E-48	6.0065874	up
Zm00001d048832	Leucine-rich repeat receptor-like protein kinase	9.48E-30	6.0080407	up
Zm00001d041922	Hypothetical protein	1.21E-42	6.0560176	up
Zm00001d043121	Osmotin-like protein OSM34	7.32E-60	6.1039559	up
Zm00001d018734	Pathogenesis-related protein PRB1-3	1.28E-60	6.3258038	up
Newgene_4421	Uncharacterized protein	1.38E-23	6.4054738	up
**Zm00001d011886**	**O-glycosyl hydrolase family**	1.05E-25	6.452923	up
Zm00001d031158	Pathogenesis related protein-5 precursor	2.30E-137	6.6822434	up
**Zm00001d006538**	**O-Glycosyl hydrolases family 17**	4.42E-67	7.1355619	up
**Zm00001d042143**	**Glucan endo-13-beta-glucosidase**	9.67E-40	7.7485877	up
Zm00001d020652	Plant viral-response family protein-like	6.19E-26	-6.4388332	down
Zm00001d045974	Polygalacturonase QRT2	3.17E-20	-6.0428287	down
Zm00001d020651	Plant viral-response family protein	1.55E-40	-5.9656011	down
**Zm00001d039958**	**Glycosyl hydrolase family 10 protein**	8.22E-43	-5.9060028	down
Zm00001d044652	Integral membrane protein like isoform X1	4.85E-18	-5.7370599	down
Zm00001d014971	Leucine-rich repeat receptor-like protein kinase	1.14E-18	-5.6846765	down
**Zm00001d052793**	**Probable beta-D-xylosidase 7**	7.10E-45	-5.6305443	down
**Zm00001d043393**	**Beta-galactosidase precursor**	6.73E-17	-5.616013	down
Zm00001d037008	Putative FAD-binding berberine family protein	3.89E-14	-5.1844361	down
Zm00001d000016	Fatty acid desaturase 4, chloroplastic	1.09E-14	-5.1806033	down

Table S3. The DEGs related to auxin synthesis and signaling.

Gene ID	Protein	WT-A	WT-B	WT-C	dnl2-A	*dnl2-B*	*dnl2-C*	Log2FC	Regulated
Zm00001d053004	AIC2	36.963974	21.803602	22.603825	4.844719	0.97185	1.426895	-3.2107702	down
Zm00001d039006	ARF3	39.114409	36.377736	32.902254	14.015454	18.157237	24.771207	-1.1532382	down
Zm00001d042809	AUX1	281.89527	218.58875	151.24978	87.724596	102.11933	132.40248	-1.2154544	down
Zm00001d049551	CYP71D10	4.883289	2.845584	4.110153	1.249599	0.323808	0.494565	-2.3892397	down
Zm00001d013302	IAA14	16.139425	19.171806	23.849813	25.42425	9.124495	11.764862	-1.0398898	down
Zm00001d010411	IAA27	88.484923	90.633244	72.271609	42.899086	46.315316	54.135498	-1.0418379	down
Zm00001d000288	IAA29	12.640019	12.000702	8.822044	2.121034	5.62152	4.834026	-1.5375461	down
Zm00001d044083	PIN10	1.44302	2.906968	4.026497	1.170158	0.273557	0.478706	-1.9835172	down
Zm00001d018024	PIN2	19.503494	21.539412	15.703957	10.331378	10.431133	11.983352	-1.0209568	down
Zm00001d018200	SAUR12	33.718307	24.964014	14.381343	5.5275	6.915839	6.439002	-2.058493	down
Zm00001d053884	SAUR50	15.975823	11.510479	17.567947	7.880973	2.146915	7.046109	-1.4607366	down
Zm00001d031146	SAUR70	26.058176	22.11865	7.280673	6.57159	8.729148	5.318528	-1.4617009	down
Zm00001d018652	YUC	40.119098	42.497759	37.858734	11.016996	7.115233	25.125542	-1.5895673	down

Zm00001d041711	ABP1	77.416256	82.809637	80.394142	205.94797	214.66507	164.14571	1.030238	up
Zm00001d013098	AOX2	3.40212	3.126399	3.895619	9.889053	13.282288	8.246822	1.2322743	up
Zm00001d015243	ARF20	4.922988	4.510323	3.415409	10.432435	21.009375	13.709839	1.5026273	up
Zm00001d023625	CYP71A	0.621067	0.454504	0.231468	1.969297	1.79293	1.946495	3.1134842	up
Zm00001d006199	CYP71A1	0.076948	0.120799	0.296593	3.647428	2.806633	1.582386	1.3943823	up
Zm00001d039310	CYP71A26	31.245235	32.057514	12.755123	70.695343	113.62765	70.973114	1.6736447	up
Zm00001d020628	CYP84A1	4.898921	4.955731	3.915683	7.722246	14.917626	16.847712	1.2001618	up
Zm00001d010697	GH3.6	0.078483	0.295179	0.177894	0.420337	7.67516	2.957535	2.874359	up
Zm00001d039345	GH3.6	1.938675	1.378186	0.967603	2.275654	7.048837	7.214149	1.501	up
Zm00001d043244	GH3.6	0	0	0	0.483522	2.118454	1.069099	4.8375757	up
Zm00001d039513	IAA2	4.673951	2.410615	1.353262	9.815515	17.536695	26.450134	2.1284263	up
Zm00001d049715	IAA25	0.202277	0.057773	0.301845	1.24675	3.015523	1.545277	2.4078138	up
Zm00001d038784	IAA26	1.852224	1.85069	0.932397	6.37507	7.00484	7.964708	1.8242002	up
Zm00001d043515	IAA6	2.727783	2.166351	0.658787	8.310275	10.507529	8.690279	1.8219972	up
Zm00001d043660	PIN8	0	0	0	3.763943	0.746595	0.655363	4.1375649	up
Zm00001d025947	SAUR11	3.678255	4.069687	2.905218	8.327682	15.064765	11.126365	1.3551989	up
Zm00001d033464	SAUR14	0.852877	1.073447	1.507344	5.939686	16.515339	12.129838	2.6075559	up
Zm00001d021459	SAUR61	0.759573	0.720663	0.881145	3.012895	26.013107	6.755065	2.7836052	up
Zm00001d026308	SAUR71	7.582423	9.784674	6.156706	17.592503	24.350832	17.352926	1.0342675	up

Table S4. The DEGs related to GA synthesis and signaling.

**Gene ID**	**Protein**	**WT-A**	**WT-B**	**WT-C**	***dnl2-C***	***dnl2-B***	***dnl2-A***	**Log_2FC**	**Regulated**
Zm00001d046342	KO	0.454307	1.193713	0.614289	2.360689	7.17858	4.072183	2.0392353	up
Zm00001d045563	DWARF3	12.115951	9.006569	12.490876	22.36342	41.772926	59.161598	1.5073941	up
Zm00001d039634	DWARF1	0.71919	1.106431	2.745754	0.125936	0.176715	0.244001	-2.685986	down
Zm00001d038165	GID1-like	12.188163	12.493366	23.262151	45.01094	36.11118	64.695629	1.2851977	up

Zm00001d034898	GA20ox1	0.933756	0.405243	0.084867	7.213039	26.274746	5.932484	3.3986134	up
Zm00001d029031	CYP714B3	2.839965	3.064059	2.398754	14.960422	15.452813	15.108593	2.0824911	up
Zm00001d017294	GA2ox4	0.354919	0.270652	0	1.129401	1.590572	8.366607	2.3379088	up
Zm00001d010308	GID1A	2.979351	4.448784	3.482451	16.966638	17.428203	22.515989	2.0303789	up

Table S5. The DEGs related to other phytohormones synthesis and signaling.

Phytohormone	Gene ID	Protein	Log_2FC	Regulated
ABA	Zm00001d025544	Zeaxanthin epoxidase	-1.294	down
	Zm00001d025545	Zeaxanthin epoxidase	-1.062	down
	Zm00001d011117	Abscisic acid 8'-hydroxylase	1.070066748	up
	Zm00001d005884	ABSCISIC ACID-INSENSITIVE 5-like protein 5	1.263970115	up
	Zm00001d040787	Abscisic stress-ripening protein	2.517046824	up
	Zm00001d023529	Abscisic stress-ripening protein	1.326584818	up
	Zm00001d026603	Abscisic acid receptor; Mg-chelatase H subunit	-1.873	down
	Zm00001d047037	PPYR/PYL-receptor	-1.997	down
	Zm00001d008530	ABA4 neoxanthin synthas	-1.802	down
	Zm00001d013568	Abscisic acid-inducible protein	1.699344386	up
	Zm00001d033623	lipoxygenase	2.184712137	up
	Zm00001d033624	lipoxygenase4	1.475429055	up
	Zm00001d042541	lipoxygenase2	2.70564393	up
	Zm00001d017418	Aldehyde dehydrogenase	-1.267549921	down
	Zm00001d025958	Aldehyde dehydrogenase	1.795828001	up
	Zm00001d004731	Aldehyde dehydrogenase	1.554682746	up
	Zm00001d044340	aldehyde dehydrogenase 5	1.554682746	up
JA	Zm00001d034382	60 kDa jasmonate-induced protein	1.67452743	up
	Zm00001d011377	Jasmonic acid-amido synthetase JAR1	1.10588714	up
	Zm00001d052468	BTB/POZ domain and ankyrin repeat-containing protein NH5.2	2.273248588	up

	Zm00001d023246	BTB/POZ domain and ankyrin repeat-containing protein NH5.2	3.124134463	up
	Zm00001d034944	BTB/POZ domain and ankyrin repeat-containing protein NPR3	1.424572739	up
	Zm00001d011878	elongated mesocotyl1	1.228680095	up
	Zm00001d006860	Protein TIFY 10B	1.151988418	up
	Zm00001d039469	TNF receptor-associated factor 21	2.162142316	up
	Zm00001d022139	uncharacterized protein LOC100276383	1.152418807	up
	Zm00001d028282	uncharacterized protein LOC100279433	1.567417673	up
	Zm00001d027900	unknown	3.032043121	up
	Zm00001d027901	ZIM-transcription factor 16	1.32480184	up
	Zm00001d038300	Putative cytochrome P450 superfamily protein	-1.664019474	down
**CK**	Zm00001d043293	cytokinin dehydrogenase 4 precursor	1.289363211	up
	Zm00001d049952	histidine-containing phosphotransfer protein3	1.084080883	up
	Zm00001d013021	isopentenyl transferase IPT5	2.431263924	up
	Zm00001d032046		1.388	up
**BR**	Zm00001d000298	BRASSINOSTEROID INSENSITIVE 1-associated receptor kinase 1	5.467553705	up
	Zm00001d007180	Cytochrome P450 714A1	1.945573561	up

Table S6. The DEGs related to cellulose synthesis.

Gene ID	Protein	WT-A	WT-B	WT-C	*dnl2-C*	*dnl2-B*	*dnl2-A*	FDR	Log_2FC	Regulated
Zm00001d047276	Brittle stalk 2	631.20526	542.302	389.28534	156.71129	119.28024	113.38105	4.30E-17	-2.18627	down
Zm00001d043477	CESA11	765.22521	650.80031	456.12546	260.2833	154.08691	145.7535	5.87E-09	-1.894354	down

Zm00001d032776	CESA10	578.06925	473.06107	343.99729	161.31225	96.30339	101.25348	3.92E-12	-2.119282	down
Zm00001d022082	COBRA-like 4	21.540026	17.787895	36.973072	13.764912	6.539729	10.443643	0.0003656	-1.457444	down
Zm00001d020531	CESA12	250.1056	198.82277	146.7052	75.985386	40.804151	39.397385	2.30E-09	-2.06925	down
Zm00001d007208	COBRA-like 7	1.846031	1.718169	1.75751	6.78847	3.376412	4.559125	0.0017606	1.147007	up
Zm00001d005775	CESA7	307.19929	243.63649	164.72948	103.71257	60.511349	55.019228	1.12E-07	-1.856215	down

Table S7. The DEGs related to xylan synthesis.

**Gene ID**	**Protein**	**WT-A**	**WT-B**	**WT-C**	***dnl2-C***	***dnl2-B***	***dnl2-A***	**FDR**	**Log$_2$FC**	**Regulated**
Zm00001d054066	GT61	173.3494	112.78406	125.57924	55.970722	49.239189	62.117596	4.19E-09	-1.505338	down
Zm00001d048608	AXY	183.49588	130.1122	77.444199	35.913689	27.344481	27.744711	1.08E-10	-2.228947	down
Zm00001d047104	GUX	0.382685	0.444823	0.173626	2.836904	1.226053	6.399569	2.50E-05	2.558686	up
Zm00001d046005	GT61	2.691415	2.930773	1.48562	7.733505	9.225128	18.921482	1.82E-05	1.9130303	up
Zm00001d043879	GUX	79.883365	71.430079	58.618139	33.06047	22.96978	21.274238	1.73E-10	-1.64393	down
Zm00001d042276	GUX	189.35852	153.80516	131.3773	84.125534	53.750401	58.32534	4.89E-08	-1.478956	down
Zm00001d041782	GXM	27.76852	17.919426	28.529949	10.445472	13.83484	12.480029	8.66E-05	-1.212083	down
Zm00001d039144	RWA	194.3944	186.17411	158.75711	111.86914	86.565157	85.958116	1.12E-08	-1.15253	down
Zm00001d038101	GT	81.727727	77.634022	98.107002	40.175433	35.763693	46.858216	1.21E-09	-1.285471	down
Zm00001d036543	IRX14	304.13904	239.85071	179.61401	119.3738	98.450508	100.58785	1.38E-07	-1.392296	down
Zm00001d032686	UXT	106.58165	82.995152	48.612483	39.066547	29.332734	32.788044	3.68E-05	-1.411107	down
Zm00001d029920	GUX	0.269267	0.121548	0.108663	0.647643	1.144049	1.433268	0.000936	2.0005997	up
Zm00001d028980	GT47	2.528714	2.435992	3.979892	1.067442	1.3418	1.564628	0.0012113	-1.279567	down
Zm00001d027646	GUX	25.436239	23.280569	30.497583	11.988972	8.228416	10.780794	2.21E-10	-1.560553	down

Zm00001d025869	UXT	61.854135	54.265086	49.573429	27.82803	19.665054	23.960037	5.29E-10	-1.441194	down
Zm00001d014965	GT	140.45001	117.9206	107.07516	36.275135	26.888241	33.913136	7.62E-23	-2.116113	down
Zm00001d014532	IRX15-like	43.91906	27.903107	32.855846	20.324692	14.170213	15.429946	9.78E-06	-1.276409	down
Zm00001d014525	GT	265.85391	196.54477	157.04076	117.78824	83.05925	92.211067	7.86E-06	-1.286503	down
Zm00001d011959	GT47	126.70885	110.45013	83.640651	40.061868	31.392138	27.909157	2.13E-14	-1.900985	down
Zm00001d010976	GT43IRX9	263.0816	206.05873	175.19316	104.12906	94.508118	95.625549	2.93E-09	-1.349767	down
Zm00001d009693	RWA	315.83783	259.04871	246.70265	124.16718	74.217979	116.6587	4.64E-09	-1.584588	down
Zm00001d007269	GXM	45.432903	37.59864	29.649399	6.273278	3.80959	6.827106	5.71E-20	-2.847997	down
Zm00001d004525	RWA	0.757188	0.629268	0.546389	1.708556	2.183966	2.494899	0.0007462	1.2792104	up
Zm00001d003012	UXT	148.56857	125.26282	150.91739	52.480434	43.682602	56.590645	3.79E-17	-1.692549	down

Table S8. The DEGs related to lignin synthesis.

**Gene ID**	**Protein**	**WT-A**	**WT-B**	**WT-C**	***dnl2-C***	***dnl2-B***	***dnl2-A***	**FDR**	**Log_2FC**	**Regulated**
Zm00001d052841	CCoA-OMT	422.71582	329.56619	480.61179	14.529776	13.349878	101.89378	0.0036918	-2.277269	down
Zm00001d052813	UGT89B2	0.173696	0.065898	0.052559	0.740403	0.628074	0.91014	0.0026474	2.0396931	up
Zm00001d052324	LAC	64.648865	43.485329	33.188629	9.836596	8.606112	9.693538	6.21E-17	-2.474908	down
Zm00001d051166	PAL4	32.941616	29.996542	17.511639	14.123204	7.121233	8.45079	2.24E-05	-1.591515	down
Zm00001d051161	PAL3	2221.5891	1638.6178	827.34366	889.26143	742.02638	503.52707	0.0003068	-1.392547	down
Zm00001d050224	4CLL1	11.561841	9.655196	9.765507	5.72089	3.8373	4.819643	1.22E-07	-1.334419	down
Zm00001d046915	4CCL4	129.2498	139.07884	116.54458	64.846745	69.241802	81.360387	1.61E-08	-1.120056	down
Zm00001d043166	UGT87A1	1.899218	1.866701	1.298588	16.534668	42.697316	20.765238	2.66E-18	3.4634107	up
Zm00001d042906	LAC17	359.45551	239.39389	185.13722	41.382977	19.960648	28.520798	1.61E-19	-3.181667	down
Zm00001d042905	LAC17	38.717171	17.656382	34.903053	12.941505	3.48384	9.327847	0.0001791	-1.862804	down
Zm00001d038371	LAC	89.679853	54.496711	38.895111	44.875084	15.944169	21.915819	0.006302	-1.306015	down
Zm00001d034069	UGT83A1	1.64571	0.821728	0.961762	4.434361	5.132932	3.155114	0.0003662	1.5047206	up

Zm00001d033320	UGT91A1	1.109671	1.57873	1.418983	2.820545	4.189148	3.399539	0.0070503	1.0561895	up
Zm00001d033055	4CLL7	10.309948	9.097127	8.761469	5.822784	6.194193	4.329389	7.56E-05	-1.005624	down
Zm00001d028599	LAC17	140.16669	114.37427	52.750813	44.583279	28.340618	17.040075	5.02E-05	-1.847216	down
Zm00001d021755	UGT88A1	1.052158	0.801945	0.98365	0.106914	0.397887	0.06671	0.0050475	-1.956441	down
Zm00001d018660	4CLL5	9.470172	6.652882	4.637338	4.62207	2.807762	2.778542	0.0032769	-1.197084	down
Zm00001d017279	PAL7	45.82972	36.143543	31.79541	15.136882	11.664035	17.521851	6.37E-09	-1.558363	down
Zm00001d015618	CAD	713.04517	491.3838	402.8161	295.45597	330.80951	255.89258	0.0003609	-1.082572	down
Zm00001d015459	4CLL3	515.64451	375.42379	276.19997	101.78088	97.402311	95.259411	2.53E-13	-2.104207	down
Zm00001d014126	UGT86A1	5.795413	4.837064	5.072094	18.405783	21.573587	10.057516	0.0001495	1.3470555	up
Zm00001d012408	LAC	53.033314	33.369274	36.557796	7.713882	2.647958	6.446166	7.59E-13	-2.886933	down
Zm00001d012144	4CLL9	1.672334	3.217074	3.82929	0.90126	0.877441	1.45198	0.0004861	-1.534977	down
Zm00001d003102	UGT92A1	10.763176	7.937758	8.026229	3.791221	3.740132	5.577008	0.000154	-1.221811	down
Zm00001d003016	PAL2	979.84337	762.46893	463.88049	166.69056	111.3417	106.48987	2.32E-15	-2.632414	down
Zm00001d003015	PAL6	69.273827	52.307652	39.678391	10.073579	7.149182	7.772137	4.22E-24	-2.832792	down

Table S9. The DEGs related to pectin and cell wall proteins.

	Gene ID	Proteins		Log_2FC	Regulated
APGs proteins	Zm00001d027471	fasciclin-like arabinogalactan protein	FLAs	-1.692535	down
	Zm00001d009567	fasciclin-like arabinogalactan protein	FLAs	-1.6470329	down
	Zm00001d034373	fasciclin-like arabinogalactan protein	FLAs	-1.4697858	down
	Zm00001d020903	fasciclin-like arabinogalactan protein 7 precursor [Zea mays]	FLAs	-2.1546924	down
	Zm00001d039770	fasciclin-like arabinogalactan protein 7 precursor [Zea mays]	FLAs	-2.2182171	down

	Zm00001d019994	fasciclin-like arabinogalactan protein 2 [Zea mays]	FLAs	-1.9109236	down
	Zm00001d010832	fasciclin-like arabinogalactan protein 11 [Zea mays]	FLAs	-2.3825873	down
	Zm00001d013108	fasciclin-like arabinogalactan protein	FLAs	-1.1226781	down
	Zm00001d034874	expressed in cucumber hypocotyls [Zea mays]	AGPs	-1.6507073	down
	Zm00001d038532	lipid binding protein precursor [Zea mays]	AGPs	-1.7942083	down
	Zm00001d034427	systemin receptor SR160 precursor [Zea mays]	AGPs	-1.9793192	down
**Pectin synthesis**	Zm00001d028824	uncharacterized protein LOC100191914 precursor [Zea mays]	GATL	-1.1995362	down
	Zm00001d047062	Ras-related protein Rab5 [Zea mays]	GATL	-1.9719988	down
	Zm00001d042514	Ras-related protein Rab5 [Zea mays]	GATL	-1.4195397	down
	Zea_mays_newGene_4578	galacturonosyltransferase 11	GATL	-1.5713994	down
	Zm00001d020986	O-fucosyltransferase		1.39921594	up
	Zm00001d015309	O-fucosyltransferase family protein [Zea mays]		1.14718988	up
	Zm00001d035899	glycoside hydrolase, family 28 precursor [Zea mays]	GH	1.98895746	up
	Zm00001d027441	glycoside hydrolase, family 28 precursor [Zea mays]	GH	-1.8009591	down
	Zm00001d039958	glycosyl hydrolase family 10 protein / carbohydrate-binding domain-containing protein [Zea mays]	GH	-5.9060028	down

Table S10. The DEGs related to cell wall loosening.

Gene ID	Annotation	Protein	Log_2FC	Regulated
Zm00001d043393	beta-galactosidase precursor	bglA	-5.616013034	down
Zm00001d032022	Beta-galactosidase	bglA	-1.635045948	down
Zm00001d048993	Putative alpha-L-arabinofuranosidase family protein, partial	ASD	-2.250722308	down
Zm00001d052425	Putative alpha-L-arabinofuranosidase family protein	ASD	-1.799562615	down
Zm00001d028952	Alpha-L-arabinofuranosidase 1	abf1	-1.165525274	down
Zm00001d034723	mannan endo-1,4-beta-mannosidase 3 isoform X2		2.547373584	up
Zm00001d019752	probable pectinesterase 15	petcin	1.531941372	up
Zm00001d053225	Beta-galactosidase	pectin	-1.852399705	down
Zm00001d010204	uncharacterized protein LOC100273392 isoform X2	GATL	-1.321435782	down
Zm00001d015292	uncharacterized protein LOC100384439 precursor	EG	-1.744797052	down
Zm00001d051814	endoglucanase 7-like precursor	EG	-1.678336102	down
Zm00001d017978	Endoglucanase 1	EG	-1.042039711	down
Zm00001d047538	Endo-1,4-beta-glucanase	EG	1.899456613	up
Zm00001d035083	expansin A24	Expansin	-4.56303232	down
Zm00001d017494	beta-expansin 4 isoform X1	Expansin	-1.24566724	down
Zm00001d029906	beta-expansin 7 precursor	Expansin	-2.364318507	down
Zm00001d047090	beta-expansin 1a precursor	Expansin	-1.136009166	down
Zm00001d047087	beta expansin8	Expansin	2.066837667	up
Zm00001d047093	expansin-B6	Expansin	-3.809236313	down
Zm00001d029783	expansin-like 3 precursor	Expansin	1.511553389	up
Zm00001d006360	expansin-like A3	Expansin	1.859788572	up
Zm00001d039251	glucan endo-1,3-beta-D-glucosidase-like precursor	glcA	-2.246875764	down
Zm00001d006538	glucan endo-1,3-beta-glucosidase 7	glcA	7.135561895	up
Zm00001d021695	glucan endo-1,3-beta-glucosidase 13 isoform X1	glcA	4.699133777	up

Zm00001d010137	Glucan endo-13-beta-glucosidase 14	glcA	1.782315136	up
Zm00001d029313	PLASMODESMATA CALLOSE-BINDING PROTEIN 5	glcA	-1.955393189	down
Zm00001d049359	glucan endo-1,3-beta-glucosidase 6	glcA	-1.902767495	down
Zm00001d007509	unknown	glcA	-1.573395708	down
Zm00001d042140	uncharacterized protein LOC100272379 isoform X1	glcA	2.441350004	up
Zm00001d011886	Putative O-Glycosyl hydrolase superfamily protein	glcA	6.452922967	up
Zm00001d053733	glucan endo-1,3-beta-glucosidase, acidic isoform-like	glcA	4.171515054	up
Zm00001d042143	glucan endo-1,3-beta-glucosidase, acidic isoform precursor	glcA	7.748587687	up
Zm00001d024891	Beta-glucosidase 17	glcA	2.81458715	up
Zm00001d025847	beta-glucosidase isoform X1	glcA	-1.406747128	down
Zm00001d037918	Non-cyanogenic beta-glucosidase	glcA	1.902391218	up
Zm00001d014489	Putative beta-glucosidase 41	glcA	-2.078569616	down
Zm00001d046210	Beta-glucosidase 11	glcA	-1.341035555	down
Zm00001d021119	Beta-glucosidase 11	glcA	1.7693523	up
Zm00001d004626	probable beta-D-xylosidase 7	XYL	-1.24324738	down
Zm00001d052793	probable beta-D-xylosidase 7	XYL	-5.630544324	down
Zm00001d047683	NAD(P)-binding Rossmann-fold superfamily protein		-1.134595372	down
Zm00001d028825	NAD(P)-binding Rossmann-fold superfamily protein		-1.811089939	down
Zm00001d029814	xyloglucan endotransglucosylase/hydrolase protein 32 precursor	XTH	1.747174577	up
Zm00001d027313	uncharacterized protein LOC100194192 precursor	XTH	-1.390153991	down
Zm00001d002446	WAK53a-OsWAK receptor-like protein kinase	WAK	1.541212539	up
Zm00001d032356	putative wall-associated receptor kinase-like 16	WAK	2.910049018	up
Zm00001d003019	Protein kinase superfamily protein	WAK	2.310950633	up
Zm00001d002447	Wall-associated receptor kinase 2	WAK	2.283640889	up
Zm00001d002979	Protein kinase superfamily protein	WAK	3.819830289	up
Zm00001d003021	wall-associated receptor kinase 5 isoform X1	WAK	2.137712928	up

Zm00001d017264	wall-associated receptor kinase 5	WAK	2.385450978	up
Zm00001d003023	putative WAK family receptor-like protein kinase isoform X1	WAK	2.359070115	up
Zm00001d028280	wall-associated receptor kinase-like 14	WAK	1.64378235	up
Zm00001d030941	hypothetical protein ZEAMMB73_Zm00001d030941	WAK	-1.679726352	down
Zm00001d050164	putative WAK-related receptor-like protein kinase family protein precursor	WAK	2.456261354	up
Zm00001d003823	cutin	cutin	1.636588198	up
Zm00001d049834	lipid transfer protein precursor	cutin	-2.462469497	down
Zm00001d031636	putative bifunctional inhibitor/LTP/seed storage protein family precursor	cutin	1.029571695	up

Table S11. Primers for qRT-PCR validation.

Gene Name	Sequence (5'-3')
Tubulin-F	CTACCTCACGGCATCTGCTATGT
Tubulin-R	GTCACACACACTCGACTTCACG
Zm00001d045563-F	AGAGCAAAGGCGGAGCA
Zm00001d045563-R	TGACGGAAGGAGACGAAGG
Zm00001d029906-F	TGCCCAACACCTACTACCGTTC
Zm00001d029906-R	CATCAGTCCCATGCAGAAATACA
Zm00001d047276-F	TGACCGTGACCGAACCAA
Zm00001d047276-R	CCAAATAAAATGCCCTGCCTAC
Zm00001d010308-F	ACACACAGACACACACACCACA
Zm00001d010308-R	ACGAGCCAAGCAAGCAGAG
Zm00001d010697-F	GGACTTCACCATGAGGCACTAC
Zm00001d010697-R	TACATCCACCATCTTCCATACCC
Zm00001d043879-F	TGACGTCGGTGGCTTCTCT
Zm00001d043879-R	CTCTTCCAGTTCATCCCCTTGT
Zm00001d052841-F	CGCCATCAGGGAACTCAA
Zm00001d052841-R	CAGAGACGACGAGCGAATG
Zm00001d004855-F	TTCATCGGAAGTGCTTCTAAG
Zm00001d004855-R	TCAACAACATTGCCTGCTC
Zm00001d034898-F	GGACTTCACCATGAGGCACTAC
Zm00001d034898-R	TACATCCACCATCTTCCATACCC
Zm00001d013039-F	CGTCTACCCGGAGAAGGTCA
Zm00001d013039-R	GCGGCGTGATGTGTGATTAG
Zm00001d039958-F	GACAGCATCGGCAACTTCAA
Zm00001d039958-R	CGTCCGCTTCTTCACCTCTC
Zm00001d035083-F	AGGGCCAGTTCTGATTCGTTT
Zm00001d035083-R	GACATGGAGTTATGGGGCAGTAG
Zm00001d043244-F	AGCAACGGCAAGGCAGA
Zm00001d043244-R	CGATACAACCCGGAGTAGGTG
Zm00001d052793-F	TCCAGAGCCAGCACATCAA
Zm00001d052793-R	TTCTGCCATCGTCCCTCAC
Zm00001d016943-F	CTTCCCTTCCAACCTCAGAAAA
Zm00001d016943-R	AGGAGGAGCACAGGATGAGG
Zm00001d005446-F	AGTCTGCTCAAGGACCCCAT
Zm00001d005446-R	TTGAGTCCACGGTGAATGAAG
Zm00001d029970-F	CCTCCTCATCACCTCCGT
Zm00001d029970-R	TGCCGAACAAGAAGTCCA
